# Ligature of the Subclavian Artery, &c.

**Published:** 1830-10-01

**Authors:** 


### XXXI.

### Case of secondary Hæmorrhage, in which the Subclavian Artery was tied above the Clavicle. By M. S. Buchanan, M.D. Surgeon of the Glasgow Royal Infirmary.*

This interesting case is detailed at great length in the last (XI.) Number of our esteemed Glasgow cotemporary; and we shall here present a concise analysis of the case.

*Case.* Will. Stewart, aged 55, was admitted on the 19th April, under the care of Dr. Weir. He had been attending a vat with boiling solution of alum; and by the fumes from which he was overpowered. He fell down, and the right hand and fore-arm dropped into the boiling lye. In this state he was found by his fellow workmen, and taken home insensible. When brought into the hospital, two days afterwards, the whole of the arm, from the elbow to the points of the fingers, seemed quite dead, the skin like boot-leather. The hand and part of the fore-arm were insensible—the pulse barely perceptible at the wrist. A consultation determined that amputation should be de-

* Glasgow Royal Infirmary.

layed, and a variety of local and general means were used, such as turpentine dressings—poultices—calomel and opium, &c.

"Two days after admission, he had some head affection, indicated by the pain of forehead, and incoherence, as well as by the contracted state of the pupil, and the partial paralysis of his left arm. There was also present another most important symptom, and which I beg your attention to, as of great consequence in all cases of extensive injuries, I mean the appearance of the skin and adnata; these were of a pale yellow colour, and remained so till the very last, and I have almost invariably found this symptom, one of the most unfavourable which in such cases can occur. Indeed so much impressed have I always been with this peculiar appearance of the countenance after traumatic inflammation, that when I now observe it, I draw the most unfavourable prognosis, and I am sorry to say it proves too frequently the correct one. Forty leeches were applied to the head, after which the cold lotion was had recourse to, the bowels were freely opened, and the other medicines continued." 234.

On the 22d the incoherency having ceased, and a line of separation having appeared, an incision was made through the whole length of the fore-arm, which was a putrid mass; and two days afterwards (24th) amputation was performed above the elbow. There was some hæmorrhage, and four arteries as well as the brachial vein were obliged to be tied. The dressings were removed on the 3d day—the flaps were found wide open—the surface brown and sloughy—the discharge offensive. Œdema had spread up the arm, and to the right side of the chest—the constitutional symptoms were not favourable, the skin still retaining the unnatural colour—the tongue dry—the stools green—pulse small and compressible. Stimulants were now given, and the stump dressed with terebinthinate applications. Half a glass of wine every hour was given. Under this treament the patient rallied considerably. On the 30th April, Dr. W. resigned, and Dr. B. took charge of the ward.

"When I took charge (says Dr. B.) this forenoon, the appearance of matters did not much please me, more particularly the hæmorrhage, for I observed that one ligature still remained, whether that around the humeral artery or vein was uncertain, and from the state of the stump, you may well understand my anxiety; I saw also, that the bedclothes and mattress were soaked with blood and discharge from the stump, and though there was a pretty good pulse remaining, yet this was all that I had to depend on. About eight o'clock in the morning of the first of May, I was summoned to a consultation, hæmorrhage having again taken place. The bed and surrounding dressings were deluged with blood, the stump of the same foul appearance, the countenance pale, the features sunk, and the extremities quite cold; in short, he was moribund. In this state the members of consultation saw him, and were of opinion that, as he had only a few minutes seemingly to live, it was quite out of the question to propose any operation. Meantime as I was most anxious that his strength should be supported, and death deferred for some time at least; in other words, that it should not be said that hæmorrhage had killed the patient, I ordered warm brandy and wine to be administered in such quantity as his stomach would bear.—He had lost, from seven o'clock, A. M. at which time the hæmorrhage had first been observed, about two pounds of blood, but at last by firmly compressing the subclavian artery, and the application of a hard pad and bandage to the brachial, high up, further loss had been prevented. I may also state that, on looking at the surface of the stump, before leaving him, I found that the last ligature was gone, probably having come away with the first gush of blood. But you remark that there was a most difficult point of practice; I had Scylla and Charybdis to avoid, I must throw in stimuli and prevent death from mortification, and if this is accomplished, and the pulse and strength rally, hæmorrhage will again undoubtedly occur. I chose the former as the safest procedure, and determined, come what might, to ward off, as long as possible, the fatal event, by the means above referred to.

Contrary to the expectation of the consultation, in the morning, this poor man recovered most wonderfully, by the visit hour, 1 o'clock, when I was again under the necessity of requesting the opinion of my seniors in attendance, and though this consultation is not recorded in the journal, I think it of considerable moment, and shall, therefore, in a few words state to you the result. A majority were of opinion that the patient should be allowed some further time to recruit, and then that the subclavian artery should be tied, and this opinion was enforced by the observation that he might lose so much blood during the performance of so hazardous an operation, that death might take place while he was on the table, an occurrence this at all times to be looked to, in any case, even the most desperate, as reflections of the very worst kind are apt to arise, however unfounded. The minority thought that the above blood-vessel should, without delay, be secured, and this last opinion was rendered the more striking, by the probability of further hæmorrhage taking place, and thus putting it out of our power altogether to move our hands; besides, it was remarked, that if this operation is performed in a dexterous and bloodless manner, then all is safe, at least on this side; and the only risk is of sinking from mortification, which can, from the man's appearance, since the morning, be avoided, by the means lately adopted; as in all such cases the opinion of the majority carried, and the same practice was steadily pursued, of supporting the strength, and of careful and constant watching.

At 6 o'clock in the evening, another consultation was called, in consequence of the pulse getting up, and also oozing to some extent having taken place from the surface of the stump, commanded, as formerly, by pressure on the subclavian. At this consultation all were agreed on the propriety of having immediate recourse to tying the above blood-vessel, but it was suggested, that before taking the knife, it might be as well to examine, with care, the surface of the sloughing stump, and see if the main artery could not be laid hold of with the tenaculum and tied. This idea, however, the moment the stump was looked at, was abandoned, and now all were decided.

Having informed the patient what was going to be done, and had all properly arranged, I turned his head a little to the left side, and requested my colleague, Dr. Perry, to depress the right shoulder, I then took my seat, and having, with my left hand, kept the skin of the neck upon the stretch, I began my incision with a common scalpel, through the skin and cellular substance, from the acromial edge of the trapezius muscle, onwards to the clavicular portion of the sterno-cleido-mastoideus, and about two lines above the clavicle. The first thing that came in my way was the external jugular vein; this I carefully drew to a side, and then cut through the platysma myoides, and superficial cervical fascia, which was here of considerable thickness. The nerves proceeding to the axillary plexus, now presented themselves, and were with ease drawn aside, the cellular substance, the connecting medium between them, being separated with the point of the finger. The immense subclavian vein was now seen, completely lying upon, and covering the artery; and though in the dead subject this vessel is found under, and to the side of the arterial tube, yet, in the living, it swells out, and causes some trouble to turn aside. This part of the operation, however, as well as the detachment of the artery from its firm connexion to the first rib, by the side of the scalenus anticus, I with great ease accomplished, with the assistance of a silver scalpel, an instrument of great use, in all operations, where delicacy of touch is required.

Having now advanced through these steps of the operation, an obstacle of some moment presented itself, to the passing of the aneurismal needle from below upwards; this was the clavicular portion of the sterno-mastoid muscle, which I was under the necessity of cutting, and then with the greatest ease the common needle was passed close by the side of the scalenus anticus, which proves so infallible a guide to the arterial tube. Having now satisfied myself, as well as all my medical friends, who had favoured me with their presence and assistance, that the subclavian, and it alone was isolated above the needle, I proceeded to tie it, and this I without difficulty accomplished, by carrying my two fingers, with the ends of the ligatures at their points, down to the bottom of the wound; a single stitch was put in the centre of the wound, and its lips brought together, with adhesive plaster, and simple dressing above. The operation did not occupy more than ten minutes; there was not so much as two tea-spoonsful of blood lost, and the pain was seemingly very trifling.

Much has been written with respect to the difficulties of this operation, and the trouble of passing the aneurismal needle from below upwards, and many have been the ingenious inventions to facilitate this part of the operation; I must confess, however, that, in my opinion, these difficulties have been sadly magnified, and here, as in most similar cases, books are of very little use, nay I think they often do harm; for if any one has dissected the parts frequently, and noted well their relative situation, I would advise him to take his own way of management, heedless of the intricate and often confused directions of systematic book-makers."

The diurnal details of the 2d, 3d, and 4th of May, we need not detail. He sunk gradually, and expired on the latter day. The inspection is short, and cannot be condensed.

"The contents of the cranium were healthy, but the *plexus choroides*, and the internal structure of the *cerebrum*, were rather paler than natural. On the right side of the chest there were strong adhesions, seemingly of long standing, and in both cavities of the pleura, more particularly on the left side, a considerable quantity of serum was effused. The substance of the lungs, the pericardium, heart, and large blood-vessels, were healthy. The abdominal and pelvic viscera were all in a healthy state, with the exception of the liver, which was considerably enlarged, and the posterior part of the right lobe of a very soft, friable texture; the gall-bladder also, and biliary ducts, were gorged with inspissated dark-coloured bile. The integuments at the anterior and right side of neck having been dissected back, the sterno-mastoid muscle, with the sterno-hyoid and thyroid cut, and turned aside, the arteria innominata was brought into view, at the giving off of the carotid; the scalenus anticus was now also cut, and turned aside, and the subclavian artery well seen, with the ligature firmly tied round it, and a hard clot of blood perceptible on its cardiac side; the clavicle was now removed, and the axillary artery traced under the pectorals, onwards to its termination in the stump, during which transit it seemed quite healthy, till about an inch below axilla, when it assumed a soft greenish appearance, and no clot could be discovered on the distal side of the ligature. The muscles surrounding the shoulder joint were soft, green, and matted together, and a large collection of fœtid pus extended from the stump, below the axilla, to the under surface of the pectoralis major and minor; the whole substance of which last, was in the same gangrenous state as the muscles of the shoulder joint."

Many judicious remarks are made on the case by the talented reporter; but we are unable to give them a place here. We quite agree with Dr. B. that death was not occasioned in this case by the last operation, but by the spreading of the traumatic gangrene, whose progress was accelerated by the hæmorrhage both during the operation and afterwards.

"But it may be asked, what was the cause of the secondary hæmorrhage on the 7th day after the amputation? I think this admits of a very satisfactory answer; not only from an inspection of the blood-vessel, but also from the colour of the blood discharged, its quantity, and its suppression, by the application of the thumb to the subclavian, as it passes over the first rib. All these circumstances show that the hæmorrhage came from the main trunk. The ligature round the blood-vessel still remains; and on inspecting its cardiac side, the hard clot of blood which nature has formed, can be traced into one of the nearest branches."

---

### XXXII.

### SURGICAL OPERATIONS PERFORMED AT THE DESIRE OF PATIENTS.

